# Evaluation of the Safety and Efficacy of Conventional Transarterial Chemoembolization (cTACE) and Drug-Eluting Bead (DEB)-TACE in the Management of Unresectable Hepatocellular Carcinoma: A Systematic Review

**DOI:** 10.7759/cureus.41943

**Published:** 2023-07-16

**Authors:** Javaria Ayyub, Karan Nareshbhai Dabhi, Namra V Gohil, Nida Tanveer, Sally Hussein, Shravya Pingili, Vijaya Krishna Makkena, Arturo P Jaramillo, Babatope L Awosusi, Tuheen Sankar Nath

**Affiliations:** 1 Internal Medicine, California Institute of Behavioral Neurosciences & Psychology, Fairfield, USA; 2 Internal Medicine, Medical College Baroda, Vadodara, IND; 3 Medicine, Kakatiya Medical College, Hyderabad, IND; 4 Research, California Institute of Behavioral Neurosciences & Psychology, Fairfield, USA; 5 Medicine, Osmania Medical College, Hyderabad, IND; 6 General Practice, California Institute of Behavioral Neurosciences & Psychology, Fairfield, USA; 7 Pathology and Laboratory Medicine, California Institute of Behavioral Neurosciences & Psychology, Fairfield, USA; 8 Surgical Oncology, Tata Medical Centre, Kolkata, IND; 9 Surgical Oncology, California Institute of Behavioral Neurosciences & Psychology, Fairfield, USA

**Keywords:** conventional transarterial chemoembolization, drug-eluting bead transarterial chemoembolization, hepatoma, hepatocellular carcinoma, liver cancer, safety, efficacy

## Abstract

Transarterial chemoembolization (TACE) is considered the preferred loco-regional treatment option for hepatocellular carcinoma (HCC) not amenable to resection due to its distinctive blend of precise drug administration, localized tumor management, and reduced systemic adverse effects, setting it apart from the plethora of alternative treatments available. There is an ongoing debate regarding the optimal choice for managing HCC using TACE, particularly between its two major types: conventional TACE (cTACE) and drug-eluting bead TACE (DEB-TACE). The medical community remains divided on which approach offers superior safety and efficacy, with conflicting evidence and varied outcomes adding to the complexity of this nuanced decision. Given the lack of consensus surrounding the preferred TACE technique in treatment-naive patients for HCC, we conducted a rigorous systematic review to assess and contrast the relative safety and efficacy of cTACE versus DEB-TACE in patients diagnosed with HCC who did not receive any prior treatment for HCC. Our study aimed to provide much-needed clarity on this controversial topic, shedding light on the two approaches' comparative safety and efficacy to inform clinical decision-making. After a comprehensive search of databases and search engines and through a methodical screening process, including standardized quality assessments and relevant filter application based on our eligibility criteria, we identified 10 articles pertinent to our research query comprising two randomized controlled trials, one meta-analysis, and seven observational studies. The collective sample size of the studies was 5,288 patients with HCC, of which 2,959 were in the cTACE arm and 2,324 were in the DEB-TACE arm.

## Introduction and background

Liver cancer contributes significantly to cancer prevalence worldwide, and it is expected to have an incidence of over one million people by 2025 [1]. In the US, it is the fourth and fifth major cause of mortality due to cancer in the age groups of 50-64 and 65-79 years, respectively. Although the incidence of liver cancer in men has remained stable in men aged 50 years and above, its incidence in women has consistently increased [2]. Common etiologies of liver cancer include cirrhosis due to chronic infection with hepatitis B virus (HBV) and hepatitis C virus (HCV), alcohol consumption, metabolic syndrome, diabetes, obesity, non-alcoholic steatohepatitis, and aflatoxins [1,3,4]. Hepatocellular carcinoma (HCC) remains the most prevalent pathological variant of cancer arising in the liver in adults [1]. This article uses the terms liver cancer, liver tumor, and HCC interchangeably.

The diagnosis of liver cancer includes mainly non-invasive testing, including imaging and blood tests, to establish the type and stage. The selection of treatment modality, whether curative or palliative, is determined by the stage of HCC, which is evaluated by the Barcelona Clinic Liver Cancer (BCLC) staging system, which outlines the treatment algorithm for HCC, taking into account the prognosis and tumor characteristics, such as the number and size of the tumor, baseline hepatic function assessed through the Child-Pugh scoring criteria, and performance status (PS) of the patient, which corresponds to their general health and fitness, to achieve the best possible outcome. For those who have liver cancer classified as very-early stage (0) or early stage (A), loco-regional treatment options, including ablative procedures using chemicals, heat, cold, microwave, or radio waves, and electrochemotherapy, resection, or liver transplant (LT) are considered. Meanwhile, systemic chemotherapy alone or in combination with surgical management or supportive care is generally administered to patients with advanced and terminal-stage HCC [5].

Transarterial chemoembolization (TACE) is the standard treatment for intermediate-stage (B) liver cancer with multi-nodular tumors, preserved liver function (Child-Pugh A-B), and PS 0. The 2022 BCLC update recommends TACE for down-staging tumors >3 cm in non-LT patients and selective TACE as a bridge to LT in patients awaiting transplants with greater than six months wait time. It has been considered the treatment of choice for unresectable HCC [5]. TACE includes conventional TACE (cTACE) and drug-eluting bead TACE (DEB-TACE). Other techniques of embolization include transarterial radioembolization (TARE) and bland embolization.

In TACE, after the feeding vessel has been identified by hepatic angiography, it is catheterized using a microcatheter. In cTACE, the chemotherapeutic drug (e.g., doxorubicin) emulsified in Lipiodol (a drug carrier) is given as an arterial infusion. The tumor-feeding artery is then embolized using polyvinyl alcohol (PVA) or a gelatin sponge. Lipiodol is a lymphographic agent extracted from poppy seed oil, which is retained preferably by the tumor. It causes cytotoxicity and ischemia and slowly releases the dissolved chemotherapeutic agent. By contrast, DEB-TACE uses microspheres as embolic agents and drug carriers. The embolization is considered complete when the occluded vessel shows no blood flow on hepatic angiography [6].

Numerous studies have examined the tolerability, treatment response, safety profile, long-term survival, possible clinical complications, and cost-effectiveness of cTACE and DEB-TACE. However, the results of these studies have been equivocal. Some evidence suggests the superiority of DEB-TACE to cTACE in treatment response, survival, and safety profiles [7]. Others have found that DEB-TACE has better tolerability than cTACE, with no difference between the two treatments in terms of overall survival (OS) [8]. Finally, a few others pointed to the association of DEB-TACE with increased clinical complications than cTACE [9]. There needs to be coherence among the results from various studies, given that some articles show striking differences in their findings. Consequently, a definitive conclusion cannot be drawn regarding the comparability of the two approaches.

This systematic review aims to address the discrepancies concerning safety in terms of the tolerability and efficacy of cTACE compared to DEB-TACE in the adult population with HCC.

## Review

Methods

This systematic review strictly adhered to the Preferred Reporting Items for Systematic Reviews and Meta-analysis (PRISMA) 2020 guidelines [10].

Search Strategy

We searched for the relevant literature in the central databases and search engines, including PubMed, MEDLINE, PubMed Central (PMC), and Google Scholar, using Medical Subject Headings (MeSH) controlled vocabulary thesaurus and keywords [11-13].

On April 2, 2023, we searched for the relevant articles on PubMed, MEDLINE, and PMC using the MeSH strategy: cTACE OR Conventional TACE OR Trans-arterial Chemo-embolization OR DEB-TACE OR Drug Eluting Beads Trans-arterial Chemo-embolization OR Chemo-embolization OR ("Chemoembolization, Therapeutic/adverse effects"[Majr] OR "Chemoembolization, Therapeutic/methods"[Majr] OR "Chemoembolization, Therapeutic/mortality"[Majr] OR "Chemoembolization, Therapeutic/standards"[Majr] OR "Chemoembolization, Therapeutic/statistics and numerical data"[Majr]) AND HCC OR ("Carcinoma, Hepatocellular/drug therapy"[Majr] OR "Carcinoma, Hepatocellular/therapy"[Majr]).

To find the pertinent articles in Google Scholar, on April 22, 2023, we used the keywords: “conventional transarterial chemoembolization,” “cTACE,” “drug eluting bead transarterial chemoembolization,” “DEB-TACE,” “HCC,” “hepatoma,” “hepatocellular carcinoma,” and “liver cancer.”

Eligibility Criteria

We included review articles, randomized control trials (RCTs), and observational studies conducted on the adult population, published in the last five years in English. Articles published in languages other than English, studies involving animal populations, or gray literature were excluded. A summary of the inclusion and exclusion criteria for this review is given in Table 1.

**Table 1:** Eligibility criteria for the studies included in this review RCT: randomized controlled trial; cTACE: conventional transarterial chemoembolizationc; DEB-TACE: drug-eluting bead TACE Table credit: Javaria Ayyub

Inclusion criteria	Exclusion criteria
RCTs, observational studies, meta-analyses, systematic reviews	Animal studies, case reports, opinion articles, letters
Articles published in the last five years	Articles published more than five years ago
Published articles	Gray or unpublished literature
Papers in the English language	Papers in a language other than English
Hepatocellular carcinoma (HCC)	Population of non-HCC patients
Studies including a direct comparison of cTACE and DEB-TACE for the treatment of HCC	Radioembolization, combination treatment with TACE, ablation, surgery, transplant, systemic treatment for HCC
Studies including patients who were treatment-naïve for HCC and received TACE as the first-line treatment	Studies including patients who had received treatment for HCC (other than TACE) after diagnosis
Studies reporting a comparison of safety and efficacy between cTACE and DEB-TACE	Studies comparing cTACE and DEB-TACE on aspects other than safety and efficacy

Study Risk-of-Bias Assessment

For the quality assessment of the articles and bias evaluation, we used (1) the Assessment of Multiple Systematic Reviews (AMSTAR) tool for the meta-analyses and systematic reviews, (2) the Cochrane risk-of-bias (RoB) assessment tool for RCTs, and (3) Newcastle-Ottawa Scale (NOS) for assessing the quality of the observational studies. To include only the high- and medium-quality articles, 11 studies were analyzed using these standard quality assessment tools, and 10 high and medium-quality articles were selected.

**Figure 1:**
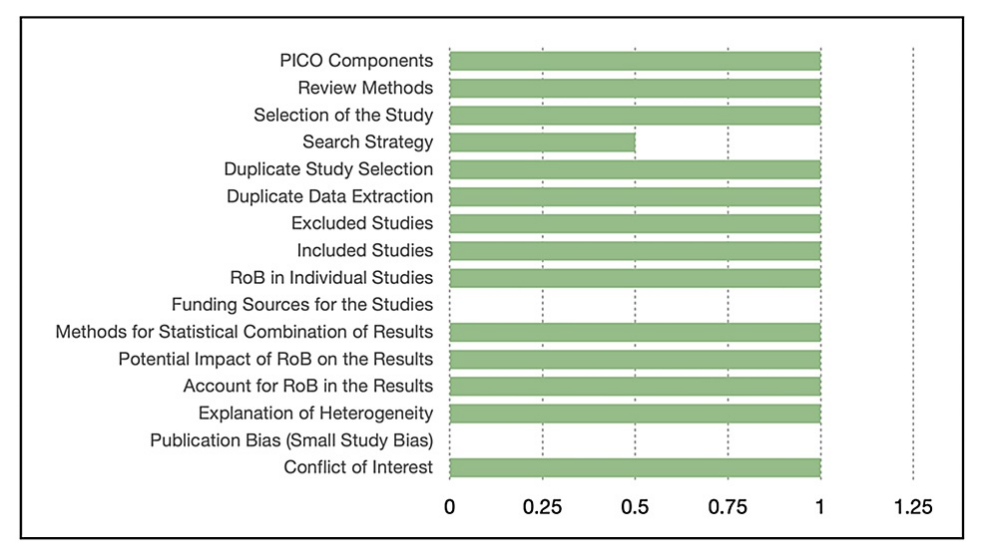
Quality check for Han et al. using the AMSTAR tool AMSTAR: Assessment of Multiple Systematic Reviews; PICO: patient/population, intervention, comparison, outcome; RoB: risk of bias Figure credit: Javaria Ayyub

**Figure 2:**
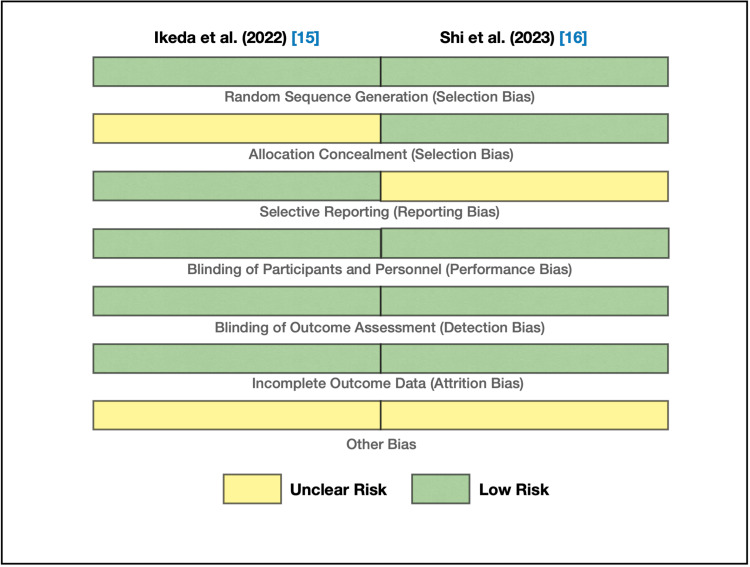
Bias assessment using the Cochrane RoB tool RoB: risk of bias Figure credit: Javaria Ayyub

**Figure 3:**
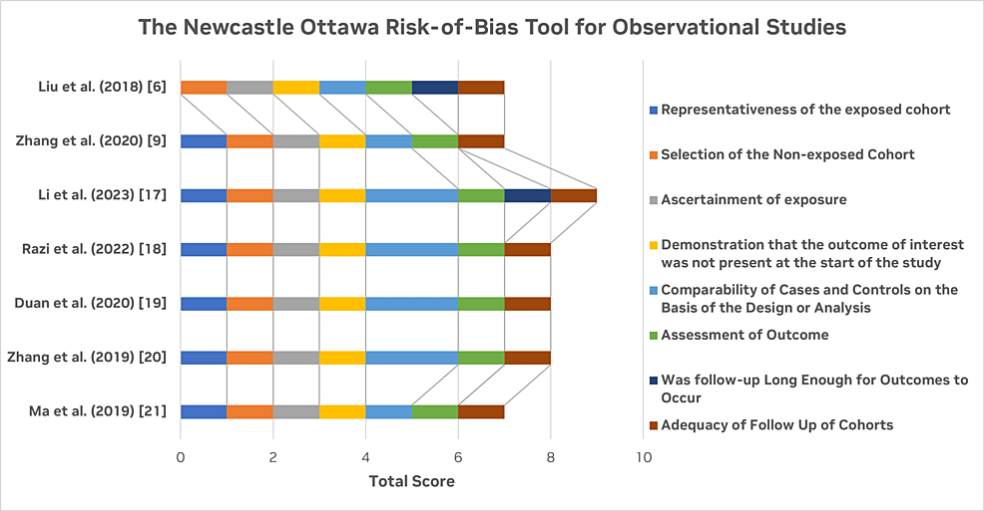
Quality assessment for the included observational studies using the NOS NOS: Newcastle-Ottawa Scale Figure credit: Javaria Ayyub

The scoring and quality assessment of the selected articles, done by two independent investigators who reached a consensus, are represented Figures 1-3. Figure 1 shows that the meta-analysis by Han et al. (2019) [14] met all the pre-defined criteria in the AMSTAR tool and got perfect scores except for the publication bias, the funding source for the study, and a partial yes and, hence, half the points in the search strategy criterion. It got a total score of 14/16, according to this tool.

We used the Cochrane RoB tool for the two randomized controlled trials: Ikeda et al. [15] and Shi et al. [16]. These studies are at low RoB, as shown in Figure 2, except for the unclear risk of allocation concealment bias in Ikeda et al., unclear risk of reporting bias in Shi et al., and an unclear risk of other bias in both studies. Accordingly, they were selected as medium- and high-quality articles, respectively.

Figure 3 shows the graphical representation of the calculated score for the quality assessment of the observational studies [6,9,17-21] using the NOS. All the selected studies are high or medium quality with a low RoB in terms of the selection of the cohorts, their comparability, and the follow-up of cohorts.

Data Extraction

Two independent reviewers collected the data from the reports using the methods mentioned earlier and confirmed the eligibility of the data to be evaluated in concordance with each other. The selected studies were carefully inspected for (1) the type of the article, (2) number of patients participating in the study, (3) the number of cTACE and DEB-TACE procedures done on the patients, (4) OS, (5) progression-free survival (PFS), (6) complete response (CR), and (7) post-embolization syndrome (PES).

Results

**Figure 4:**
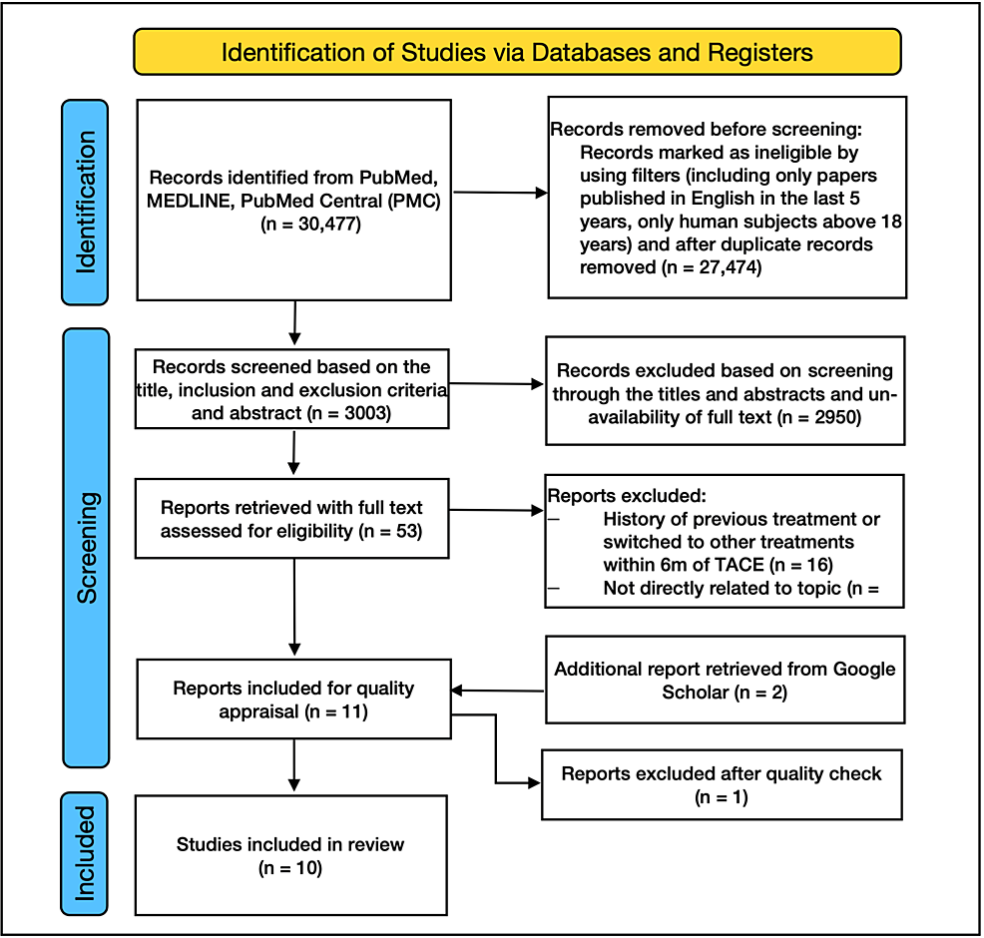
PRISMA 2020 flow diagram for systematic reviews PRISMA: Preferred Reporting Items for Systematic Reviews and Meta-analysis Figure credit: Javaria Ayyub

From our initial search of PubMed, MEDLINE, and PMC, we found 30,477 articles. We removed 27,474 articles based on the eligibility criteria. We screened the rest of the 3,003 articles by their titles. Except for 53 articles, all were excluded due to the unavailability of full text and topics about treatments other than TACE. Of the 53 articles, 16 were discarded because, in those studies, the included patients with HCC had a history of previous treatment for HCC or they switched to other forms of therapy within six months of the intervention, and 28 were excluded because they were not directly related to our research. We additionally identified two more studies from Google Scholar, and they were then assessed for quality appraisal in addition to the nine final studies. Of these 11, one low-quality article was discarded. The PRISMA flow diagram for the 10 selected studies is shown in Figure 4 [10].

Of the 10 included articles, seven were observational studies, two were RCTs, and one was a meta-analysis. They directly compared the safety and efficacy of cTACE versus DEB-TACE in 5,288 treatment-naïve patients with HCC. Of these, 2,959 patients underwent cTACE, and the remaining 2,324 underwent DEB-TACE. The baseline patients’ characteristics were comparable in almost all the included studies.

Discussion

HCC usually arises in patients with chronic cirrhosis, and surgical resection is the preferred treatment option, yielding a five-year survival rate of about 60%. However, only up to 15% of HCC patients can undergo surgery due to many individual factors, such as age, PS, stage of the disease, and comorbid conditions [14]. On imaging, HCC usually shows high vascularity on the arterial phase (AP). On the venous phase (VP), it displays wash-out, making it possible to induce ischemia and necrosis of tumor cells by embolizing the vessels supplying the tumor with an anti-cancer drug. As a result, TACE is the standard treatment approach for HCC not amenable to resection or for the down-staging of the disease. There are two main types of TACE: cTACE and DEB-TACE. Both procedures are performed in digital subtraction angiography (DSA) rooms and commonly use the femoral approach to perform celiac trunk angiography. If the tumor vasculature does not appear abundant with the celiac trunk approach, an investigation of the extrahepatic source of the vasculature, including the superior mesenteric artery (SMA), inferior phrenic artery, internal mammary artery (IMA), right adrenal, or right inferior intercostal artery, is conducted [16]. Portal vein patency and hepatic artery anatomy are also assessed during the procedure. After selective angiography, the microcatheter is guided through the hepatic artery to the segmental or subsegmental vessels supplying the tumor to ensure superselective chemoembolization.

cTACE is performed by injecting a solution of drug carrier (ethiodized oil called Lipiodol) and chemotherapeutic drug (usually doxorubicin or epirubicin) into the vessel, which is then embolized using a gelatin sponge or PVA slurry to prevent back leakage of the drug. Due to the pharmacokinetics of cTACE involving the delivery of small-sized Lipiodol-drug emulsion, it can be done to very small-sized vessels, including collaterals that may develop after the embolization. However, there are several concerns regarding this process, including that the Lipiodol and the drug are not bound to each other chemically by a bond. Hence, they separate very quickly, resulting in the flow of the drug into the systemic circulation, accounting for its reduced therapeutic benefit on the tumor tissue and its broader side effect profile. Moreover, Lipiodol is very embolic, and it damages the capillaries resulting in the inflammation of the peribiliary plexus and liver capsule. It causes complications, such as intrahepatic biloma, thrombus formation in the portal vein, infarcts in the liver, severe liver failure, and sepsis, leading to mortality. cTACE also causes more periprocedural complications, such as ischemic pain, bradycardia, and derangements in blood pressure. In addition, the embolic agents used in cTACE include gelatin and PVA, which come in various shapes and sizes; therefore, a complete distal embolization can be challenging [18]. Finally, Lipiodol causes halos on the imaging; hence, the tumor's size on the follow-up of patients after cTACE could not be accurately measured [15].

By contrast, DEB-TACE uses DEB beads, CalliSphere microspheres, or HepaSpheres as the drug carriers and embolic agents. These beads release the anti-cancer drug very slowly and consistently, targeting only the cancerous tissue. Embolization is considered complete when the blood flow stops up to near-stasis in the tumor-supplying vessel. DEB-TACE offers permanent super-selective complete embolization of the vessel supplying the tumor; therefore, it spares the surrounding normal liver parenchyma from toxicity. This results in increased delivery of the chemotherapeutic agent to the tumor, thereby reducing drug concentration in the general circulation. Hence, it has a lower risk of side effects arising from systemic circulation. The biliary plexus that supplies the bile ducts originates from the hepatic artery. As doxorubicin levels in the local tissue are very high, there is an increased incidence of bile duct dilation because doxorubicin is known to cause hepatic artery stenosis. Bile duct dilation is the most frequently occurring side effect of TACE [15]. Some studies report that periprocedural pain is vague and tolerable, with no significant changes in the vital signs. However, compared to cTACE, which uses very small-sized embolic agents, the beads used in DEB-TACE are relatively bigger. They cannot be used to embolize the very fine collaterals that develop after TACE. Therefore, DEB-TACE cannot be repeated in this case [18].

Safety of cTACE versus DEB-TACE

TACE can have adverse effects evident both clinically and on laboratory tests. The most common side effect is PES, which constitutes fatigue, pyrexia, nausea and vomiting, abdominal pain and distention, hiccups, loss of appetite, constipation, hypoalbuminemia, hyperbilirubinemia, and an increase in aminotransferase enzymes. Systemic toxicity manifests in patients as granulocytopenia, bone marrow toxicity, increased international normalized ratio (INR), thrombocytopenia, or ascites after TACE [9]. To evaluate the safety of TACE, liver function tests (LFTs) are measured at baseline before the procedure and are then regularly followed up until a predefined period after TACE. For HCC patients with cirrhotic liver, the reservation of liver function becomes an important priority.

The study done retrospectively on 192 HCC patients by Ma et al. in 2019 [21] to provide the comparison of the safety and efficacy of the two types of TACE noted that the DEB-TACE group had an increased incidence of peri-interventional pain (p=0.037), post-interventional pain (p=0.035), and pyrexia (p=0.023) compared to the cTACE group during the hospital stay, which can be attributed to rapid tumor necrosis and possible hepatic artery damage caused by DEB-TACE. The effect of cTACE and DEB-TACE on the LFTs was similar at M0 and M1 after the procedure. However, the baseline characteristics of both groups show a statistically significant contrast in tumor distribution, total bilirubin, and Eastern Cooperative Oncology Group (ECOG) PS with more unifocal tumors, increased bilirubin levels, and poor ECOG scores in those who underwent DEB-TACE. It also shows that the patients in the DEB-TACE group responded better to the treatment than the cTACE group [21].

In concordance with the results noted by the study mentioned above, a multicenter retrospective study done by Zhang et al., in 2020 [9], on 1,002 patients (608 with cTACE and 394 with DEB-TACE treatment) to record clinical complications on imaging after cTACE versus DEB-TACE also documented a higher frequency of biliary dilatation (p<0.001) and portal vein narrowing (p=0.006) in the DEB-TACE group. In addition, there was an increased incidence of liver failure (p=0.03) and severe pain in the abdomen (p<0.001) in the DEB-TACE group than in the cTACE group. It is, however, noteworthy to mention that there was a statistically significant difference in the BCLC stage (p=0.01) and age between the patients in the two groups with older patients (p<0.001) in the DEB-TACE as compared to cTACE [9].

In contrast to the findings mentioned above, a randomized controlled trial conducted in 2022 by Ikeda et al. [15] on 200 patients with HCC recorded a higher incidence of PES, including pyrexia (p=0.0001), malaise (p=0.01013), loss of appetite (p=0.0048), and elevated transaminases: aspartate transaminase (AST) and alanine transaminase (ALT) (p<0.0001), hyperbilirubinemia (p=0.0002), and hypoalbuminemia (p=0.0154) in patients who underwent cTACE than those with DEB-TACE. It should be noted that, in this study, cTACE has shown a better response to the treatment than DEB-TACE [15]. Endorsing the safety of DEB-TACE is the conclusion of the study done by Razi et al. in 2022 [18], which included 40 patients in a retrospective study with 20 patients in the cTACE group and 20 patients in the DEB-TACE group. Compared to DEB-TACE (15%), there was a higher incidence of intractable refractory pain in cTACE (90%) during the procedure. cTACE was observed to have a slightly higher incidence of PES, elevated LFTs, and worsening of the Child-Pugh class [18].

Liu et al. (2018) [6] conducted a retrospective study on five-year outcomes of cTACE versus DEB-TACE in 273 HCC patients. It noted that the patients with DEB-TACE had lower mean AST and bilirubin, and the percentage of patients with normal AST and bilirubin was higher in the DEB-TACE group than in the cTACE group. It also showed a decreased mean ALT in patients in the DEB-TACE group. However, there was no significant difference in the percentage of patients with normal ALT in either groups, demonstrating that DEB-TACE could be more beneficial than cTACE in terms of the long-term treatment outcomes of HCC patients [6]. Finally, a meta-analysis by Han et al. in 2019 [14], which included 167 studies accounting for 3,195 patients, of which 1,746 patients were in the cTACE group and 1444 patients in the DEB-TACE group, concluded that there was no significant difference in terms of safety of cTACE and DEB-TACE [14].

A summary of the studies comparing the safety of cTACE against DEB-TACE is shown in Table 2:

**Table 2:** Summary of collected data regarding the procedural safety of cTACE and DEB-TACE from the studies in this review cTACE: conventional transarterial chemoembolization; DEB-TACE: drug-eluting bead TACE; HCC: hepatocellular carcinoma; RCT: randomized controlled trial; LFT: liver function test; AST: aspartate transaminase; ALT: alanine transaminase Table credit: Javaria Ayyub

Authors [publication year]	Study type	Number of patients	Intervention studied	Results	Conclusions
Ma et al. (2019) [21]	Retrospective cohort	192; cTACE (n=98), DEB-TACE (n=94)	Comparison of cTACE and DEB-TACE for HCC in terms of safety	Patients receiving DEB-TACE experienced more pain during the intervention (p=0.037), pyrexia (p=0.023), and pain (p=0.035) during their hospital stay.	DEB-TACE patients experienced more adverse effects during the procedure and hospital stay.
Zhang et al. (2020) [9]	Retrospective cohort	1002; cTACE (n=608), DEB-TACE (n=394)	Complications of the intervention cTACE vs. DEB-TACE in HCC	DEB-TACE was associated with abdominal pain following the intervention than cTACE, with a statistical significance of p<0.001. In addition, DEB-TACE demonstrated higher rates of bile duct dilation (p<0.001), portal vein narrowing (p=0.006), and liver failure (p=0.03) against cTACE.	Weighed against cTACE, DEB-TACE is linked to an increased possibility of experiencing pain, liver toxicity, and injury to the liver or biliary system.
Ikeda et al. (2022) [15]	RCT	200; cTACE (n=101), DEB-TACE (n=98)	Assessment of adverse effects in the two types of TACE for HCC	A higher incidence of adverse effects (including pyrexia, pain, loss of appetite, and derangements in LFTs) was reported in cTACE patients compared to DEB-TACE patients.	cTACE had a higher incidence of adverse effects compared to DEB-TACE.
Razi et al. (2022) [18]	Retrospective cohort	40; cTACE (n=20), DEB-TACE (n=20)	Side effects of cTACE vs. DEB-TACE in early-stage HCC	DEB-TACE resulted in slightly more severe procedural complications, such as peritumoral ischemia and bile duct dilation. By contrast, cTACE had a higher incidence of refractory pain (90%) during the intervention than DEB-TACE (15%), along with a slightly higher incidence of post-embolization syndrome (PES), an increase in Child-Pugh class, and elevated LFT levels after the intervention.	DEB-TACE was noted to have more intra-procedural and immediately post-procedural complications.
Liu et al. (2018) [6]	Retrospective cohort	273; cTACE (n=201), DEB-TACE (n=72)	Comparison of conventional and DEB-TACE in patients with HCC five years after the intervention	Patients with DEB-TACE had lower mean AST, ALT, and bilirubin. The percentage of patients with normal AST and bilirubin was higher in the DEB-TACE group than in the cTACE group.	DEB-TACE showed better long-term outcomes as compared to cTACE.
Han et al. (2019) [14]	Meta-analysis	3195; cTACE (n=1746), DEB-TACE (n=1444)	Comparison of safety of cTACE compared to DEB-TACE in unresectable HCC	No significant difference between either groups in terms of safety.	Safety in either groups is comparable.

Considering these results, we concluded that DEB-TACE might show increased side effects immediately after the procedure due to the complete and permanent occlusion of the vessel supplying the tumor. However, in the long term, its safety is comparable to cTACE in treating patients with HCC. The choice of intervention should be made considering many factors, including the patient’s age, BCLC stage, ECOG PS, Child-Pugh class, physician’s judgment, patient preference, and tumor morphology and blood supply.

Efficacy of cTACE and DEB-TACE in Terms of Survival

Their effect on long-term survival measures the efficacy of these procedures. This is calculated by PFS, which denotes the period from the intervention to the time that the progression of the disease can be measured radiologically, and by OS, which is calculated as the interval of time between the initial embolization procedure and the patient’s mortality due to any cause.

A randomized controlled trial on 90 patients by Shi et al. in 2023 [16] observed an increased OS of patients in the DEB-TACE group with 534 days as compared to 367 days in the cTACE group (p=0.027) and a longer average PFS in DEB-TACE of 352 days versus 278 days in the cTACE group (p=0.004) [16]. A meta-analysis by Han et al. in 2019 [14] involving 3195 patients with HCC, with 1746 patients who had cTACE treatment and 1444 patients who had DEB-TACE treatment, concluded that three-year survival was significantly increased in the DEB-TACE group in comparison to that of the cTACE group with statistically significant heterogeneity [14].

In 2023, a retrospective cohort by Li et al. [17], which enrolled 121 HCC patients, showed the effect of cTACE (59 patients) compared to DEB-TACE (62 patients) on hepatic fibrosis in intermediate and advanced HCC. The study showed prolonged PFS in the DEB-TACE group with a median survival of 10 months versus six months in cTACE (p<0.001) and an increase in OS in DEB-TACE with an average survival of 21 months compared to 16 months in cTACE (p=0.003). However, neither PFS nor OS differed in either treatment groups in severe hepatic fibrosis. Compared to cTACE, DEB-TACE showed an increase in PFS (p=0.004) in patients with non-severe fibrosis of the liver, whereas the OS did not have any significant difference between either groups [17].

Another interesting approach was adopted by Duan et al. in 2020 [19], who prospectively conducted the study using an uncommonly used chemotherapeutic agent, arsenic trioxide (ATO), comparing 48 patients with cTACE to 38 patients with CalliSphere beads loaded with ATO (CBATO), which is a form of DEB-TACE. The results showed a longer PFS of 308 days in the CBATO arm compared to 148 days in the cTACE arm. It also showed an increase in OS in the DEB-TACE (CBATO) group, 548 days compared to 404 days in the cTACE group. It also concluded that TACE with CBATO is an independent factor in predicting a better response to treatment. However, this study used an unusual anti-cancer, so the results might be challenging to generalize [19].

In 2019, a retrospective cohort by Ma et al. [21], which enrolled 192 patients for HCC, concluded that there was no significant difference in the PFS or OS between the two groups. However, it showed that an increased AFP (p=0.038), Child-Pugh Class B or C (p=0.006), and the largest tumor size >7cm (p=0.004) independently predicted worse PFS in HCC patients. However, due to the contrast in baseline patient characteristics in both the groups and the short follow-up duration, the data may need to be more adequate to calculate PFS and OS and make deductions on this basis [21].

The retrospective study showing the five-year outcome of 273 patients with cTACE (201 patients) against those of DEB-TACE (72 patients) for HCC done by Liu et al. [6] in 2018 noted that a more significant percentage of patients in the cTACE arm (76.1%) than the DEB-TACE arm (66.7%) died over the five-year follow-up (p=0.045). All of them had disease progression at the end of the five-year follow-up, but the time to progression was significantly different in these groups: 11 months in the case of DEB-TACE compared to 16 months after cTACE (p=0.019). However, the median survival time was 37 months in both the cTACE and DEB-TACE groups. Moreover, there was a significant difference in a few of the baseline demographics of the cTACE group, including age (p=0.009), Cancer of the Liver Italian Program (CLIP) stage (p=0.018), and BCLC stage A (p=0.040), compared to those in the DEB-TACE group [6].

A comparison of the two procedures in terms of their effect on survival is summarized in Table 3.

**Table 3:** Summary of studies comparing cTACE and DEB-TACE in terms of survival RCT: randomized controlled trial; CSM: CalliSphere microspheres; cTACE: conventional transarterial chemoembolization; DEB-TACE: dug-eluting bead TACE; HCC: hepatocellular carcinoma; OS: overall survival; PFS: progression-free survival; CBATO: CalliSphere beads loaded with arsenic trioxide *CBATO-TACE is a form of drug-eluting (CalliSphere) beads that use arsenic trioxide as the anti-cancer chemotherapy drug Table credit: Javaria Ayyub

Authors [publication year]	Study type	Number of patients	Intervention studied	Results	Conclusions
Shi et al. (2023) [16]	RCT	90; cTACE (n=45), DEB-TACE (n=45)	cTACE vs. DEB-TACE using CSM in HCC	DEB-TACE provided better OS than cTACE (534 days versus 367 days, p=0.027) and average PFS (352 days versus 278 days, p=0.004).	DEB-TACE patients showed survival improvement over cTACE.
Han et al. (2019) [14]	Meta-analysis	3195; cTACE (n=1746), DEB-TACE (n=1444)	Comparison of efficacy of cTACE compared to DEB-TACE in unresectable HCC	DEB-TACE had a higher survival rate at three years (p=0.049) with significant heterogeneity. Both noted no substantial change in OS or survival at one or two years.	DEB-TACE was reported to have a more remarkable three years survival but no significant difference in OS.
Li et al. (2023) [17]	Retrospective cohort	121; cTACE (n=59), DEB-TACE (n=62)	Effect of cTACE compared to DEB-TACE on hepatic fibrosis in HCC	In comparison to cTACE, DEB-TACE resulted in better PFS (p<0.001) and OS (p=0.003). Among patients with non-severe hepatic fibrosis, DEB-TACE led to a prolonged PFS (p=0.004), but OS did not differ significantly. However, neither OS nor PFS varied in treating patients with severe hepatic fibrosis.	DEB-TACE showed a reduction in the progression of hepatic fibrosis.
Duan et al. (2020) [19]	Retrospective cohort	86; cTACE (n=48), CBATO-TACE* (n=38)	Comparison of efficacy of cTACE compared to CBATO-TACE* in unresectable HCC using arsenic trioxide	CBATO-TACE had superior rates of PFS (p=0.044) and OS (p=0.021).	CBATO-TACE demonstrated higher rates of PFS and OS as compared to cTACE.
Ma et al. (2019) [21]	Retrospective cohort	192; cTACE (n=98), DEB-TACE (n=94)	Comparison of cTACE and DEB-TACE for HCC in terms of survival profile	No substantial differences were found in OS or PFS.	DEB-TACE treatment had no notable differences in overall and progression-free survival compared to cTACE.
Liu et al. (2018) [6]	Retrospective cohort	273; cTACE (n=201), DEB-TACE (n=72)	Comparison of conventional and DEB-TACE in survival in patients with HCC five years after the intervention	The mortality rate of patients was more in the cTACE group (76.1%) than in the DEB-TACE arm (66.7%) in a five-year follow-up (p=0.045). No considerable difference was seen in the average survival time in both groups.	DEB-TACE showed better long-term outcomes as compared to cTACE.

Our analysis of the studies comparing cTACE to DEB-TACE regarding survival in terms of PFS and OS shows that DEB-TACE is, at the least, comparable to cTACE in treatment-naïve patients for HCC and chosen appropriately according to the patient profile and tumor characteristics; it might even offer a better long-term survival profile than cTACE.

Efficacy of cTACE Compared to DEB-TACE in Terms of Response to Treatment

The topic of much research is comparing the treatment response to treatment to the two types of TACE. Treatment response is usually evaluated at a predefined period after the intervention using the Modified Response Evaluation Criteria in Solid Tumors (mRECIST). The criteria for assessing the response to treatment is usually subdivided into the following classes: (1) CR, which suggests that the tumor enhancement in the AP completely disappears in response to the treatment; (2) partial response (PR), which implies that there is a decrease in tumor enhancement vasculature in the AP, thereby hinting to the reduction of the size of the tumors; (3) stable disease (SD), which indicates that there was no significant change in the lesions and the net effect neither qualified for PR or progressive disease (PD); (4) PD, which means that there is an increase in the arterial enhancement resulting from an increase in the tumor size. The response to treatment is also calculated by the objective response rate (ORR), which equals the sum of the CR and PR, and disease control rate (DCR), which equals the sum of the CR, PR, and SD. 

A randomized controlled trial by Ikeda et al. in 2022 [15], which included 200 patients with HCC in a multicenter, unblinded, prospective study with 101 patients who underwent cTACE and 99 patients who underwent DEB-TACE, showed significantly higher CR at M1 (84.2%) and M3 (75.2%) in the cTACE group versus DEB-TACE group (35.7% at M1 and 27.6% at M3) (p<0.0001). The study noted a slightly higher percentage of patients who underwent sub-segmental embolization and concluded that selective cTACE is associated with an increased CR than selective DEB-TACE [15].

By contrast, another randomized controlled trial by Shi et al. [16] in 2023, which recruited 90 patients with HCC and randomly divided them into cTACE and DEB-TACE treatment, observed a higher ORR in the DEB arm versus the cTACE arm (p=0.031) at M1 with no significant difference in CR and DCR. At M3, there was an increased ORR (p=0.003), CR (p=0.036), and DCR (p=0.025) and lower PD (p=0.025) in the DEB-TACE group compared to the cTACE group. At M6, no considerable difference was noted in the CR in both the groups except for a higher ORR (p=0.002) and DCR (p=0.031) and reduced PD (p=0.031) in beads-TACE than cTACE [16].

A meta-analysis of 167 studies, which included 3,195 patients (cTACE: 1746 and DEB-TACE: 1444) by Han et al. [14], in 2019, in contrast to the RCT mentioned above, showed that out of these studies, two showed an increase in CR in the DEB-TACE group as compared to cTACE (p=0.0048) without any significant heterogeneity. It noted that eight studies showed a higher DCR with DEB-TACE (p=0.0082) than cTACE with evident statistical heterogeneity. Thirteen studies reported an increased ORR in patients with DEB-TACE (p=0.0001) versus those with cTACE, with significant statistical heterogeneity caused by the difference in the response evaluation criteria. Fourteen studies noted a higher OS; specifically, the three-year survival in DEB-TACE (p=0.049) exceeded that in the cTACE, with significant statistical heterogeneity [14].

The prospective study using ATO comparing the safety profile and treatment efficacy of cTACE (48 patients) against DEB-TACE (38 patients) by Duan et al. [19] in 2020 documented at M3 an increased complete (p=0.025) and objective response (p=0.010) in the CBATO group (DEB-TACE using CBATO) with no substantial difference in DCR in comparison to the cTACE group. At M6, there were higher CR (p=0.001), ORR (p=0.012), and DCR (p=0.001) in the CBATO arm than in the cTACE arm. However, because ATO is not commonly used for HCC, these results might be challenging to apply [19].

Zhang et al. [20], in 2019, compared cTACE to DEB-TACE retrospectively in 89 patients with infiltrative HCC, supporting the efficacy of DEB-TACE (85.7%) to cTACE (66.7%) in prolonging DCR at M1. At M3, there was no significant difference in ORR or DCR in both groups except in subjects with portal shunt, in which DEB-TACE showed an improved DCR compared to cTACE (p=0.036). However, this was attributed to the portal shunt that resulted in poor Lipiodol retention in the tumor because it went through to the portal vein [20].

The retrospective study by Ma et al. [21] (2019), which enrolled 192 treatment-naïve patients for HCC, showed an increased ORR (p=0.002) in the DEB-TACE arm against the cTACE at M1 with no notable difference in CR and DCR. At M3, the DEB-TACE arm showed an increased ORR (p=0.005) and CR (p=0.002) compared to the cTACE group, with no substantial difference between the two treatment modalities in DCR. At M6, ORR still showed an increase with the DEB-TACE (p=0.003) as opposed to the cTACE, and no significant difference was noted in CR and ORR of either group. It should, however, be noted that in this study, compared to the cTACE arm, the DEB-TACE arm had a high ratio of unifocal tumors (p=0.047), worse ECOG performance scores (p=0.017), and increased median bilirubin concentration (p=0.003). The study also indicated that multivariate logistic regression with the forward stepwise method showed that at M1, DEB-TACE (p=0.023) and ECOG PS>1 (p=0.030) were independent factors associated with an increased ORR in HCC patients [21].

The data collected from the articles concerning the comparison of the response to treatment are summarized in Table 4.

**Table 4:** Summary of the studies in this systematic review comparing the efficacy of cTACE and DEB-TACE for HCC in terms of the treatment response RCT: randomized controlled trial; HCC: hepatocellular carcinoma; cTACE: conventional transarterial chemoembolization; DEB-TACE; drug-eluting bead TACE; CR: complete response; ORR: objective response rate; CSM: CalliSphere microspheres; DCR: disease control rate; M1: one month; M3: three months; M6: six months; SD: stable disease; TR: treatment response; APS: arterio-portal shunt; LFTs: liver function tests; PD: progressive disease; CBATO: CalliSphere beads loaded with arsenic trioxide; PR: partial response *CBATO-TACE is a form of drug-eluting (CalliSphere) beads that use arsenic trioxide as the anti-cancer chemotherapy drug. Table credit: Javaria Ayyub

Authors [publication year]	Study type	Number of patients	Intervention studied	Results	Conclusions
Ikeda et al. (2022) [15]	RCT	200; cTACE (n=101), DEB-TACE (n=98)	Assessment of CR rates between the two types of TACE for HCC	Those who underwent cTACE showed better outcomes in terms of CR at M1 (84.2%) and M3 (75.2%) compared to DEB-TACE (35.7% at M1 and 27.6% at M3).	cTACE showed higher rates of CR compared to DEB-TACE.
Shi et al. (2023) [16]	RCT	90; cTACE (n=45), DEB-TACE (n=45)	cTACE vs. DEB-TACE using CSM in HCC	DEB-TACE had a significantly improved ORR than cTACE at M1(p=0.031), M3 (p=0.003), and M6 (p= 0.002) and a higher CR at M3 (p =0.036).	DEB-TACE had a superior response to intervention to cTACE.
Han et al. (2019) [14]	Meta-analysis	3195; cTACE (n=1746), DEB-TACE (n=1444)	Comparison of efficacy of cTACE compared to DEB-TACE in unresectable HCC	DEB-TACE had a higher CR rate (p=0.0048) with no significant heterogeneity; it showed better DCR (p=0.0082), ORR (p=0.011) with substantial heterogeneity.	DEB-TACE was noted to have greater CR, DCR, and ORR.
Duan et al. (2020) [19]	Retrospective cohort	86; cTACE (n=48), CBATO-TACE* (n=38)	Comparison of efficacy of cTACE compared to CBATO-TACE* in unresectable HCC using arsenic trioxide	CBATO-TACE had better treatment response than cTACE using arsenic trioxide, as observed by the better treatment response at M3 and M6 (p<0.05).	CBATO-TACE demonstrated improved treatment response as compared to cTACE.
Zhang et al. (2019) [20]	Retrospective cohort	89; cTACE (n=33), DEB-TACE (n=56)	Efficacy of cTACE compared to DEB-TACE in infiltrative HCC (iHCC)	The study found no notable difference in ORR in both groups at one-month (M1) and three-month (M3) marks. In the short term, DEB-TACE resulted in a higher disease control rate (DCR) at M1. In HCC with portal shunt, DEB-TACE was associated with a better DCR for tumors >10 cm and those with APS at M1 (p=0.030) M3 (p=0.036) compared to cTACE.	For iHCC, DEB-TACE had a slightly higher DCR for APS and large-sized tumors.
Ma et al. (2019) [21]	Retrospective cohort	192; cTACE (n=98), DEB-TACE (n=94)	Comparison of cTACE and DEB-TACE for HCC in terms of response to the intervention	DEB-TACE treatment resulted in significantly better ORR, measured by CR and PR, at M1 (p=0.002), M3 (p= 0.005), and M6 compared to cTACE (p<0.05), along with higher incidence of SD at those same intervals (p<0.05).	DEB-TACE treatment resulted in better TR.

Therefore, in light of the results from these studies, we analyzed that DEB-TACE shows a non-inferior efficacy to cTACE for treating patients with HCC. The treatment modality should be selected keeping in mind the baseline patient characteristics and tumor morphology to ensure a personalized treatment plan for every treatment-amenable candidate with HCC.

Limitations

It is crucial to recognize the constraints and limitations of this systematic review and exercise prudence when interpreting the outcomes. First, two RCTs were in the 10 articles in this review. Based on our eligibility criteria, we included only papers in the English language in the last five years, including only the human population. Moreover, the methodology of performing TACE and the interpretation of results radiologically may differ in each study, making it difficult to generalize the results.

## Conclusions

This systematic review aimed to put forward a comparison of the safety and efficacy of cTACE and DEB-TACE in the management of patients with unresectable HCC who did not receive any previous treatment for HCC. The reviewed studies suggest that the safety profiles regarding tolerability, as evidenced by the adverse effect profile of cTACE and DEB-TACE, are similar, and the choice of intervention should be individualized, considering patients' characteristics and tumor morphology. Nonetheless, regarding the efficacy of the intervention, the analyzed data indicate that DEB-TACE is superior to cTACE in treating HCC and may provide better survival and treatment response outcomes if chosen appropriately, keeping in mind the patient characteristics. In short, efficacy can be maximized for a particular case if the choice of approach is individually tailored. However, these findings have their foundation established on a restricted count of articles. There remains a need for additional RCTs genuinely representative of the HCC population with extended follow-up periods to develop robust guidelines for selecting the most appropriate intervention.
